# Annotations

**Published:** 1898-11-19

**Authors:** 


### ANNOTATIONS.

### The Rehabilitation of Sherry.

THE *Lancet* Analytical Commission has taken a trip to Spain, and as the result of its investigations has given a high character to all such produce of the grape as has the right to the name of sherry. Everything about it seems to be good—at least, if we except the interesting but slightly unpleasant description of the wine-press. The wine is the unadulterated produce of the grape; in fact, grapes are so plentiful that it would not pay to adulterate it with anything else. Even when it is required to be sweetened, and a certain amount of "dulce" is added, this "dulce" is itself a concentrated grape liquor; while if colouring matter is required recourse is had not to vulgar burnt sugar, but to the colouring matter produced by "caramelisation" during the concentration by boiling of the grape sugar. Even the fortification of sherry by the addition of alcohol is good, for does not this alcohol also come from grapes? None of your corn spirit in sherry land, for wine spirit can be produced so cheaply and in such quantities that corn spirit is quite a superfluity. As for the addition of sulphate of lime, that is merely put in to aid in the development of the true character of sherry and in the etherification of its alcohol. So all is good; and "it cannot be correctly said that sherry in any particular contains a single ingredient foreign to its composition," which, as a cautious encomium, may fairly be said to be pretty good also. Now comes the difficulty. When you ask for sherry see that you get it! Just as our mouth was watering for some good prime *Lancet* sherry a gentleman from East Twickenham writes to the *Lancet* to say "you would add very much to the value of your work on sherry if you would trace the wine from its importation at our docks to its arrival on our tables," and goes on to say that he has friends "in the trade," whom he has questioned as to the adventures of the wine between the docks and the dinner table. These gentlemen "in the trade" all assure him that "blending" is essential and universal, and that this process of "blending" consists of the addition of "water, spirits, curacoa, burnt sugar, &c., and variously mixing different casks of sherry." This is disappointing.

### The Lady Practitioner and Her Sex.

ALTHOUGH during recent years there has been a considerable manufacture of female practitioners, they have not as yet become such common objects in this country as seriously to interfere with the comfort of the doctors of the rougher sex in the exercise of their profession. Nay, we fancy that most medical men extend to them a kindly sort of friendship and protection, looking upon them as if they were tender creatures engaged in a somewhat risky experiment, a position, by-the-bye, which they do not at all aspire to. In America, however, things are somewhat different. The doctress is no rarity, and her male competitors are beginning to detect her little weaknesses, one of which is said to be that as a competitor she makes use of what they consider an unfair weapon—namely, sex. A writer in the New York *Medical Record* says that most doctresses use their "femininity" as part of their stock-in-trade, and that this is the crux of the whole matter. Of course, no one objects to competition, but the competition must be one of knowledge and skill and of power over disease; and "when the accident of sex is included in the category of professional attainments, and flaunted in the eyes of the public as a claim to a heaven-born right to treat the diseases of women and children more skilfully than a brother practitioner can, an earnest protest must be entered." By all means let us protest if such a claim is made, and if it is asserted that woman, because she is a woman, can treat women better than a man can do. But if it be merely asserted that a woman doctor is more acceptable to certain women than a man doctor, although we may doubt the fact, we can see no good in protesting against her urging such claims as on the ground of "propriety" she may possess. In fact, as a mere abstract proposition, we should be quite inclined to agree that there is much both in surgical and in nursing work which often strains one's sense of delicacy and sexual propriety, and that the cry of "women for women" might have much in its favour. But in actual life things take a different aspect. Both surgeon and nurse are absorbed in their work, and other feelings are submerged in the interest the work involves; and, for the patient—well, people who lay down abstract propositions little reck how overwhelming is the desire for health and restoration which affects those who are ill. It is all very well to lay down strict professional rules, but the woman who is stricken with serious disease cares not whether her doctor is white or black, whether he is gruff or smooth of tongue, whether he is acting professionally or unprofessionally, or even whether what he does is right or wrong, so only that he can cure her. And this is just what people forget when they talk about "proprieties," professional or otherwise.

### The Dangers of Electricity.

IT is possible that in the not very distant future accidents due to electric currents may become much more common than they have hitherto been, and that with the yearly increasing tension of the current used the injuries met with may approximate more to those which accompany lightning-stroke than has so far been the case. In a lecture by Mr. W. H. Preece, F.R.S., delivered at the Institution of Civil Engineers, it was stated that in the transport of electricity the economical distance is limited only by the voltage which insulation can resist, and that 40,000 volts are being practically used in Utah in transmitting 2,000 horse-power thirty-two miles. This is a far greater tension than anything we are accustomed to here. That we in England shall before long have to accommodate ourselves and our manufactures to the new conditions imposed by the possibility of transporting power over long distances there can be no doubt, and when that time arrives we must be prepared to meet with new kinds of injuries produced by the enormous voltage which will then have to be employed. It is clear that England cannot afford to stand still in this matter, and also that we have no reason for so doing. We have heard much lately about Niagara as a source of electrical energy, but Mr. Preece says that electrical energy can be generated on a coalfield, where coal of good calorific power can be raised at a cost of 3s. per ton, cheaper than by a waterfall, even at Niagara.

